# Acute Exercise Induced Mitochondrial H_2_O_2_ Production in Mouse Skeletal Muscle: Association with p^66Shc^ and FOXO3a Signaling and Antioxidant Enzymes

**DOI:** 10.1155/2015/536456

**Published:** 2015-03-22

**Authors:** Ping Wang, Chun Guang Li, Zhengtang Qi, Di Cui, Shuzhe Ding

**Affiliations:** ^1^School of Physical Education and Sports Science, Hangzhou Normal University, Hangzhou 311121, China; ^2^National Institute of Complementary Medicine, University of Western Sydney, Penrith, NSW 2751, Australia; ^3^Key Laboratory of Adolescent Health Assessment and Exercise Intervention, Ministry of Education, East China Normal University, Shanghai 200241, China; ^4^College of Physical Education and Health, East China Normal University, Shanghai 200241, China

## Abstract

Exercise induced skeletal muscle phenotype change involves a complex interplay between signaling pathways and downstream regulators. This study aims to investigate the effect of acute exercise on mitochondrial H_2_O_2_ production and its association with p^66Shc^, FOXO3a, and antioxidant enzymes. Male ICR/CD-1 mice were subjected to an acute exercise. Muscle tissues (gastrocnemius and quadriceps femoris) were taken after exercise to measure mitochondrial H_2_O_2_ content, expression of p^66Shc^ and FOXO3a, and the activity of antioxidant enzymes. The results showed that acute exercise significantly increased mitochondrial H_2_O_2_ content and expressions of p^66Shc^ and FOXO3a in a time-dependent manner, with a linear correlation between the increase in H_2_O_2_ content and p^66Shc^ or FOXO3a expression. The activity of mitochondrial catalase was slightly reduced in the 90 min exercise group, but it was significantly higher in groups with 120 and 150 min exercise compared to that of 90 min exercise group. The activity of SOD was not significantly affected. The results indicate that acute exercise increases mitochondrial H_2_O_2_ production in the skeletal muscle, which is associated with the upregulation of p^66Shc^ and FOXO3a. The association of p^66Shc^ and FOXO3a signaling with exercise induced H_2_O_2_ generation may play a role in regulating cellular oxidative stress during acute exercise.

## 1. Introduction

Skeletal muscle has the remarkable ability to adapt to changes in cellular environmental influences during exercise [1]. Studies have shown that muscle stimulation can induce diverse metabolic and morphological adaptations, which are important mechanisms for controlling skeletal muscle phenotype changes [2]. For example, studies have shown that resistance exercises caused muscle hypertrophy and increased muscle strength [3], while endurance exercises increased muscle oxidative capacity [4]. Even a single bout of exercise can induce various effects including metabolic improvement [5]. Although the exact mechanism of exercise induced skeletal muscle adaptation remains to be elucidated, it has been shown that such adaptation involves a complex interplay between signaling pathways and downstream regulators, leading to specific molecular and cellular responses [6].

One mechanism affecting exercise related skeletal muscle phenotype changes involves reactive oxygen species (ROS), in particular hydrogen peroxide (H_2_O_2_) related redox activity [7]. H_2_O_2_ is a major component of ROS, generated during mitochondrial respiration. Electron leakage at specific redox centres during mitochondrial electron transfer chain reactions has been shown to be responsible for generating a significant fraction cellular ROS [8]. As a by-product of oxidative metabolism, H_2_O_2_ has certain damaging effects on cellular components such as DNA, proteins, and lipids in pathological conditions [9]. For example, Haycock et al. [10] reported that mitochondrial proteins such as succinate dehydrogenase and cytochrome oxidase showed particular susceptibility to H_2_O_2_, which induced mitochondrial dysfunction and oxidative damage in skeletal muscle. On the other hand, exercise induced ROS/H_2_O_2_ production in skeletal muscles has been shown to cause modification of mitochondrial signaling pathways [11, 12]. There is now genetic and biomolecular evidence indicating that ROS generation in mitochondria can also be finely controlled to play an important role in a wide variety of physiological processes by regulating signal transduction, gene expression, and redox reaction [13]. Among these, p^66Shc^ has been shown to orchestrate mitochondrial redox signaling by acting as a ROS sensor to regulate its redox function within mitochondria [14, 15]. p^66Shc^ is a peculiar protein, acting specifically in the mitochondrion as a redox enzyme that generates H_2_O_2_ by sequestering electrons from the respiratory chain [16]. It regulates cellular H_2_O_2_ content through changes in H_2_O_2_ purification ability, membrane oxidase activity, and mitochondrial respiratory chain proton leak, so that levels of intracellular H_2_O_2_ maintain homeostasis in mammalian cells [17]. Studies in p^66Shc^-deficient fibroblast and endothelial cells have found a remarkable resistance of these cells to exogenous oxidative stress and ROS-induced apoptosis [18]. p^66Shc−/−^ mice appeared to be protected from oxidative stress-induced apoptosis, diabetic renal damage, and age-dependent increase in emotionality and pain sensitivity [19, 20]. p^66Shc^ has been proposed to control oxidative stress response in mammals [21]. Further studies found that p^66Shc−/−^ mice showed resistance to a number of oxidative stress-related pathological damages, such as ischemia/reperfusion injury, vascular injury and atherosclerosis, hind limb ischemia, and alcohol-related liver damage [22–24]. However, it is still not very clear about the relationship between p^66Shc^ and exercise induced H_2_O_2_ generations.

Mammalian cells have a sophisticated system for scavenging ROS to nontoxic forms to defence cells against oxidative stress induced by high levels of ROS. This antioxidant defence system is composed of antioxidant enzymes such as superoxide dismutase (SOD) and catalase [25] and certain transcription factors, such as Forkhead box O3a (FOXO3a) (also named forkhead rhabdomyosarcoma-like 1, FOXO3a). FOXO3a is a member of the FOXO family proteins, which has been implicated in the regulation of oxidative stress in diverse physiological processes including stress resistance and metabolism [26]. It has been demonstrated that FOXO3a reduced ROS generation by the transcriptional activation of SOD [27] and catalase [28]. The general antioxidant effect of FoxOs has been shown in its capacity to extend life-span in nutrient-restricted organisms (such as* Caenorhabditis elegans*) and organism survival under various environmental stress [29]. However, the relationship between FOXO3a and H_2_O_2_ generation and antioxidant enzymes in exercise has not been reported.

Previous studies indicate that the physiological actions of H_2_O_2_ depend on its cellular concentrations. At low concentrations it affects cell proliferation and differentiation [6]; at higher concentrations it has been shown to induce certain antioxidant genes [30], but at pathological concentrations, it can cause cell damage or cell death [27]. Thus, it is important to understand the regulation of intracellular H_2_O_2_ production and related signaling pathways, including antioxidant enzyme activities during exercise. However, currently the molecular mechanism of endogenous H_2_O_2_ generation and its relationship to antioxidant cellular signaling pathways in skeletal muscles during exercise still remains to be elucidated, in particular regarding the relationship between acute exercise induced H_2_O_2_ generation and relevant signaling pathway. In this study we aim to investigate the association of H_2_O_2_ generation with p^66Shc^ and FOXO3a signalling and antioxidant enzymes in acute exercise. We hypothesise that p^66Shc^ is involved in the regulation of H_2_O_2_ production during acute exercise. We have investigated how acute exercise affected H_2_O_2_ production and the expression of p^66Shc^ and FOXO3a genes and proteins and their relationship to activity of antioxidant enzymes (SOD and catalase) in skeletal muscles of ICR/CD-1 mice.

## 2. Materials and Methods

### 2.1. Animal Groups

Eight-week-old male ICR/CD-1 mice were purchased from the Shanghai SLAC Experimental Animal Centre (Shanghai, China). Mice were fed on a standard chow diet and housed in a standard pathogen-free environment under controlled temperature (21 ± 3°C) and light (12 : 12 h light-dark cycle) at East China Normal University Animal Facility. Animals were acclimatised for a week before being randomly assigned to one of the following experimental groups: sedentary control (control, *n* = 6) and acute exercise for different period (45, 90, 120, and 150 min, respectively, *n* = 6 in each group). The use of animals and all experimental procedures were approved by the Experimental Animal Care and Use Committee at East China Normal University (SCXK 2007-0005). Experiments were performed in accordance with the Guidelines for the Use of Laboratory Animals published by the People's Republic of China Ministry of Health.

### 2.2. Exercise Protocol

The acute incremental exercise model used in this study was performed according to an established animal model described in previous studies [31]. Briefly, ICR/CD-1 mice from the exercise group were accustomed for 3 days of training on a treadmill. In the training (familiarisation) period, mice ran for 15 min at 8.2 m/min at a 0° incline on day 1; 15 min at 8.2 m/min at a 0° incline followed by another 15 m/min at a 5° incline on day 2; 15 min at 15 m/min at a 5° incline followed by another 15 min at 19.3 m/min at a 10° incline on day 3. After the familiarisation period, mice were subjected to an exercise program according to the following, with the first load (0°, 8.2 m/min, 53% VO_2max⁡_), then the second load (5°, 15 m/min, 64% VO_2max⁡_) and the third load (10°, 19.3 m/min, 76% VO_2max⁡_) exercise, respectively, each for 15 min, until reaching the preset 45 min, 90 min, 120 min, and 150 min. During the exercise, tails of mice are stimulated by brush to ensure mice run at preset speed and incline.

### 2.3. Tissues and Mitochondria Isolation

After completing the exercise program, mice were sacrificed rapidly by cervical dislocation. Gastrocnemius muscles and musculus quadriceps femoris were dissected. Left gastrocnemius muscles were used for mitochondrial isolation. Other muscles were immediately frozen in liquid nitrogen and then stored at −80°C. The frozen tissues were used for protein content determination, real-time PCR, and Western blotting.

Mitochondria were isolated as described previously [32, 33]. Briefly, muscles were homogenized in precooled isolation buffer (0.075 M sucrose, 0.225 M sorbitol, 1 mM EGTA, 0.1% fatty acid-free bovine serum albumin, and 10 mM TrisHCl, pH 7.4, 4°C) (1 mL buffer per 100 mg tissue). Homogenate was centrifuged at 1200 ×g for 10 min at 4°C using a Beckman centrifuge (Avanti J-26XP). The supernatant fraction was decanted and saved. The pellet was washed once with 2 volumes of isolation buffer. The supernatant fractions were combined and centrifuged at 9,000 ×g for 10 min at 4°C. The mitochondrial pellet was washed and centrifuged twice at 15,000 g for 2 min at 4°C with isolation buffer. Mitochondrial protein content was assayed using BSA (bovine serum albumin) as a standard according to Bradford. Freshly isolated mitochondria were used immediately for measuring H_2_O_2_, SOD and catalase activity assays.

### 2.4. Determination of Mitochondrial H_2_O_2_ Content

The hydrogen peroxide content in the skeletal muscle mitochondria was measured by* colorimetric* method as previously described [12], using a commercial kit, based on the reaction with molybdic acid (Jiancheng Biotech Inc., Nanjing, China). Adduct was measured spectrophotometrically at 405 nm in a plate reader (TECAN infinite M200, USA) in strict accordance with manufacturer's instructions.

### 2.5. Expression of p^66shc^ and FOXO3a mRNAs

The expression of p^66Shc^ and FOXO3a mRNAs was determined by quantitative real-time PCR (qPCR), as previously described [34]. Briefly, total RNA was extracted and purified by a commercial kit (Trizol, Invitrogen, Chromos, Singapore). Double-stranded cDNA was synthesised from 1 *μ*g of total RNA using ReverTra Ace qPCR RT Kit (Toyobo Co., Ltd, Osaka, Japan). Real-time PCR was conducted using Toyob SYBR-green PCR kit (Toyobo) and the StepOne Real-Time PCR System (Applied Biosystems, Foster City, CA, USA). PCR was performed in a fluorescence temperature cycler containing 0.8 *μ*L upstream and downstream primers, respectively; 4 *μ*L ddH_2_O, 3.0 *μ*L template; and 10 *μ*L 2.0x Master SYBR green I (containing Taq DNA polymerase, reaction buffer, dNTP mix, SYBR green I dye, and 10 mmol/L MgCl_2_) in a total volume of 20 *μ*L. Amplification occurred over a three-step cycle (denaturation at 95°C for 15 s, annealing at 61°C for 30 s, and extension and data collection at 72°C for 45 s) for 35 cycles. p^66Shc^ FOXO3a mRNAs were standard against that of *β*-actin. Relative expression level of each sample was calculated according to formula 2^−ΔCt^. Primers pairs were designed based on GenBank reference sequences and synthetized by Shanghai Sangon Biological Technology Co. Ltd, with the following sequences (5′ to 3′), forward primer: caactctaagttccctttca, reverse primer: gctgctgtacccaatcccac (p^66Shc^ [35]), forward primer: taggctgcactggggggtaa, reverse primer: actgatcagagctacaagac (FOXO3a [36]), forward primer: tgttaccaactgggacgaca, reverse primer: ctatgggagaacggcagaag (*β*-actin).

### 2.6. Expression of p^66shc^ and FOXO3a Proteins

The expression of p^66Shc^ and FOXO3a proteins was determined by Western blotting as described previously [34]. Briefly, frozen muscles (muscles from two individual animals in each group, combined into one sample) were homogenised (1 : 9 w/v) in ice-cold buffer (20 mM HEPES, pH 7.4, 100 mM KCl, 50 mM *β*-glycerophosphate, 50 mM NaF, 1 mM dithiothreitol, 0.5 mM Na_3_VO_4_, 0.2 mM EDTA, 0.1 mM phenylmethylsulfonyl fluoride, and 1 mM benzamidine). Homogenates were centrifuged for 5 min at 1000 ×g at 4°C, and then supernatant was centrifuged at 10,000 ×g at 4°C for 10 min, resolved in SDS buffer, and boiled for 5 min at 100°C [34]. The protein content was quantified using bicinchoninic acid assay with bovine serum albumin as the standard (Shanghai Sangon, Shanghai, China). Equal amounts of protein (30 *μ*g/lane) were electrophoresed in 12% SDS-polyacrylamide (120 V; Bio-Rad, Hercules, CA, USA), and proteins were transferred (1 h, 1.2 mA/cm^2^, Criterion blotter; Bio-Rad) onto a polyvinylidenedifluoride membrane. After Ponceau S staining and destaining, the membranes were blocked in 5% nonfat dry milk powder (Shanghai Sangon) in Tris-buffered saline, containing 0.1% Tween 20 (TBST) for 1 h at room temperature. Thereafter, a 1 : 200 dilution of the primary specific antibody (p^66Shc^ : sc-1695, p-FOXO3a-ser253 : sc-34894, Santa Cruz Biotechnology) in 5% TBST was added and incubated overnight at 4°C on a shaker. After the membranes were washed three times for 10 min each in 5% TBST, membranes were incubated with a 1 : 2000 dilution of the horseradish peroxidase-conjugated secondary antibody (Santa Cruz Biotechnology) in 5% TBST for 1 h at room temperature. Membranes were washed three times in TBST for 10 min each [37]. Visualisation of the reaction bands was performed with 3,3′-diaminobenzidine staining (Shanghai Sangon) and scanned densitometrically. Quantification was performed with a gel image processing system (GIS-2008; Tanon, Shanghai, China). GAPDH was used to standardize amounts of protein loaded.

### 2.7. Activity of SOD and Catalase

The total SOD activity (U/mg protein) in mitochondria of the left gastrocnemius muscles of ICR/CD-1 mice was measured using a commercial kit (Jiancheng Biotech Inc., Nanjing, China) in strict accordance with instructions. The adduct was measured spectrophotometrically at 550 nm with a plate reader (TECAN infinite M200, USA).

Similarly, the activity of catalase (U/mg protein) in mitochondria of the left gastrocnemius muscles of ICR/CD-1 mice was measured using a commercial kit (Jiancheng Biotech Inc., Nanjing, China), which is based on the reaction of ammonium molybdate with H_2_O_2_ to form a light yellow complex compound. Adduct was measured spectrophotometrically at 405 nm with a plate reader (TECAN infinite M200, USA) in strict accordance with instructions.

### 2.8. Statistical Analysis

Data were expressed as the means ± standard error of the means (SEM).  Statistical analysis was performed using SPSS21. Data were analysed by one-way analysis of variance (ANOVA), followed by Tukey post hoc tests for multiple comparisons. Correlation was analysed by linear regression, and correlation coefficient (*r*) and* P* value were calculated. For all tests the significance level was set at *P* < 0.05 or *P* < 0.01.

## 3. Results

### 3.1. Mitochondrial H_2_O_2_ Content in Skeletal Muscles of ICR/CD-1 Mice with Acute Exercise

**Figure 1:**
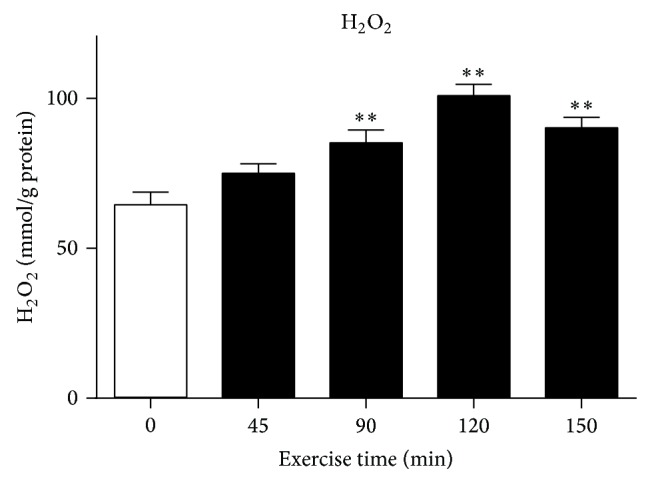
Mitochondrial H_2_O_2_ content in skeletal muscles of ICR/CD-1 mice subjected to acute exercise for different periods. Data are presented as mean ± SEM (*n* = 6). ^**^ *P* < 0.01 compared to the sedentary control group (C).

The acute exercise did not cause a significant change in the mitochondrial H_2_O_2_ content in gastrocnemius muscles of ICR/CD-1 mice with 45 min exercise, compared with that of the sedentary control. However, acute exercise caused a significant increase in mitochondrial H_2_O_2_ content in mice with 90, 120, and 150 min exercise, respectively, compared to the sedentary control (*P* < 0.01, Figure 1). The effect was time-dependent with the maximal peak effect at 120 min exercise (Figure 1).

### 3.2. Expression of p^66shc^ and FOXO3a Transcript Genes in Skeletal Muscles of ICR/CD-1 Mice with Acute Exercise

**Figure 2:**
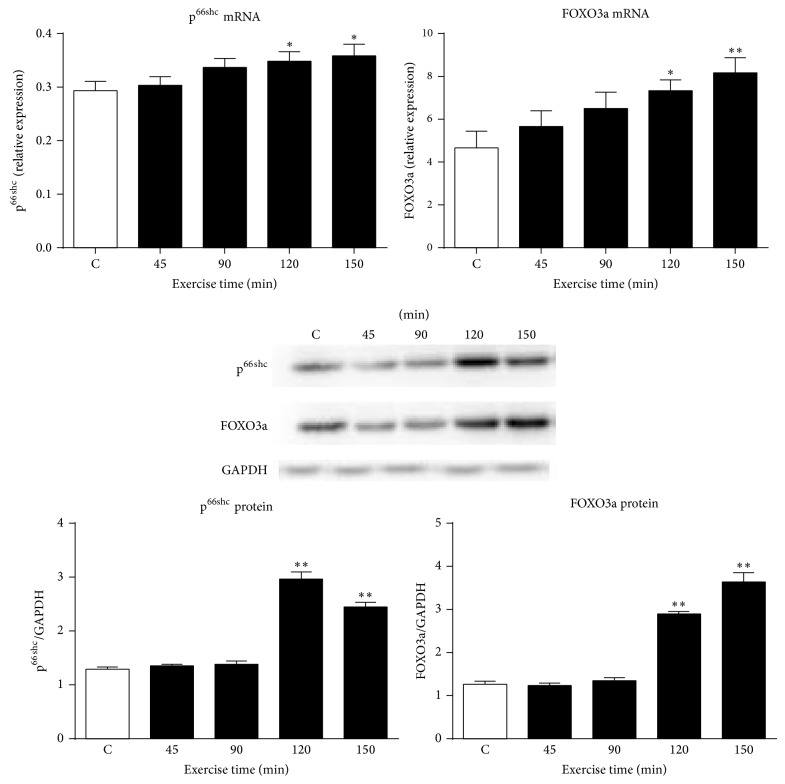
Expression of p^66Shc^ and FOXO3a mRNA and proteins in skeletal muscles of ICR/CD-1 mice, subjected to varying periods of acute exercise. Data are presented as mean ± SEM (*n* = 3–6). ^*^ *P* < 0.05, ^**^ *P* < 0.01 compared to the sedentary control group (C).

The expressions of p^66Shc^ and FOXO3a transcript genes were not significantly changed in the skeletal muscles of ICR/CD-1 mice with 45 min and 90 min acute exercise. However the expression of p^66Shc^ mRNA was significantly increased in mice with 120 min and 150 min exercise, respectively, compared to the sedentary control (*P* < 0.05, Figure 2). Similarly, the expression of FOXO3a mRNA was significantly increased in groups with 120 and 150 min exercise, compared to the sedentary control (*P* < 0.05, *P* < 0.01, Figure 2).

### 3.3. Expression of p^66shc^ and FOXO3a Proteins in Skeletal Muscles of ICR/CD-1 Mice with Acute Exercise

The expressions of p^66Shc^ and FOXO3a proteins in the skeletal muscles of ICR/CD-1 mice were not significantly changed in groups with 45 min and 90 min acute exercise (Figure 2). However the expressions of p^66Shc^ proteins significantly increased in mice with 120 min and 150 min exercise, compared to the sedentary control (*P* < 0.01, Figure 2). Similarly, the expression of FOXO3a protein was significantly increased in mice with 120 min and 150 min exercise, respectively (*P* < 0.01, Figure 2), compared to the sedentary control. The maximal effect was observed with 120 min exercise for p^66Shc^ proteins and 150 min exercise for FOXO3a proteins (Figure 2).

### 3.4. Correlation between H_2_O_2_ Content and Expressions of p^66shc^ and FOXO3a mRNA in Skeletal Muscles of ICR/CD-1 Mice with Acute Exercise

**Figure 3:**
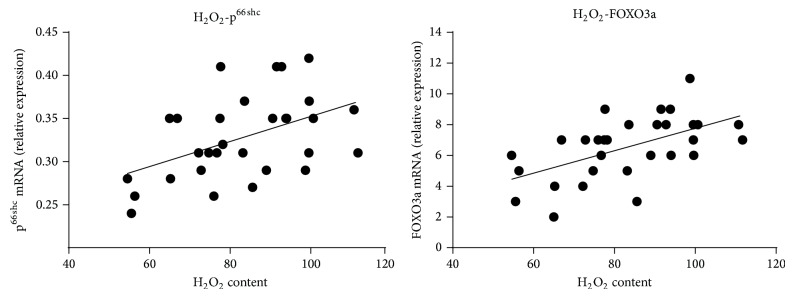
Correlation between expression of p^66Shc^ and FOXO3a mRNA and mitochondrial H_2_O_2_ content (mmol/g protein) in skeletal muscles of exercised ICR/CD.

There was a positive correlation between mitochondrial H_2_O_2_ content and expression of p^66Shc^ mRNA (*r* = 0.4723, *P* < 0.01, Figure 3) in skeletal muscles of exercised ICR/CD. Similarly, there was a positive correlation between mitochondrial H_2_O_2_ content and expression of FOXO3a mRNA (*r* = 0.5623, *P* < 0.01, Figure 3) in skeletal muscles of exercised ICR/CD-1 mice.

### 3.5. Mitochondrial SOD and Catalase Activities in Skeletal Muscles of ICR/CD-1 Mice with Acute Exercise

**Figure 4:**
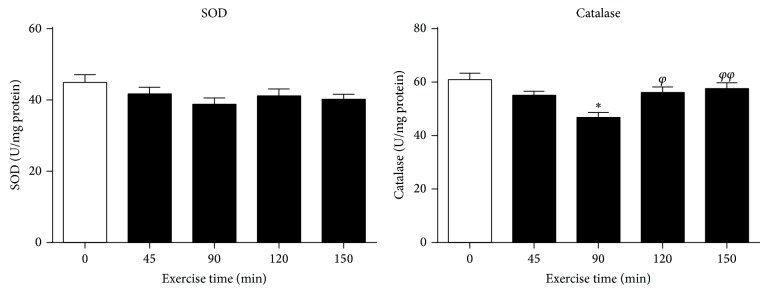
Mitochondrial SOD activity and catalase activity in skeletal muscle of ICR/CD-1 mice subjected to acute exercise over different periods. Data are presented as mean ± SEM (*n* = 6). ^*^ *P* < 0.05 compared to the sedentary control group (C). ^*φ*^ *P* < 0.05, ^*φφ*^ *P* < 0.01 compared to the 90 min exercise group.

The SOD activity was not significantly changed in groups with 45, 90, 120, and 150 min acute exercises, compared to the sedentary control (*P* > 0.05, Figure 4). The catalase activity was slightly reduced in groups with 90 min acute exercise, compared to the sedentary control (*P* < 0.05, Figure 4). However, the catalase activity was significantly higher in mice with 120 and 150 min exercise, compared to that of 90 min exercise group (*P* < 0.05, *P* < 0.01, Figure 4).

## 4. Discussion

It has been known that ROS generated during mitochondrial respiration in muscle after exercise can regulate endogenous antioxidant defence genes through the activation of redox-sensitive transcription factors [13]. For example, PGC1a, a transcriptional coactivator of genes involved in mitochondrial respiration and biogenesis and regulating antioxidant defence genes [38], can be transiently induced by endurance exercise [39]. ROS has also been linked to an increase in muscle-produced cytokines and release Ca^++^ from the sarcoplasmic reticulum during moderate exercise [40]. Among different species of ROS, H_2_O_2_ has recently been recognised as an important physiological regulator, key to regulating biological processes such as mitochondrial antioxidant defence [27]. The involvement of H_2_O_2_ in endurance exercise induced changes in signaling pathways in skeletal muscles has been recently reported for PLA2 [9]. However, the exact mechanism which links H_2_O_2_ generation and antioxidant signaling in skeletal muscle during acute exercise still needs to be elucidated. The main finding of the present study is that the acute exercise induced a time-dependent increase in mitochondrial H_2_O_2_ content, which was associated with an increased expression of p^66Shc^ and FOXO3a genes and proteins, and a time-dependent change in mitochondrial catalase activities, which highlights an interplay of mitochondria H_2_O_2_ production and modulation of p^66Shc^ signaling, and antioxidant enzyme activity induced by acute exercise in mouse skeletal muscle.

Previous studies have demonstrated that H_2_O_2_ generated during exercise can cause cell signalling change, inducing alterations in gene expression by directly modifying target proteins, or by changing their intracellular redox state [13], such as via modulation of signaling pathways for growth factors and myokine production [41]. In the present study, we observed that acute exercise caused a time-dependent increase in mitochondrial H_2_O_2_ content in skeletal muscles of ICR/CD-1 mice over the 45–150 min exercise period, with the maximal effect seen after 120 min exercise. In addition, the expression of p^66Shc^ and FOXO3a genes and proteins in skeletal muscles of ICR/CD-1 mice showed a similar time-dependent manner to that of H_2_O_2_ content change, indicating a role of p^66Shc^ and FOXO3a in H_2_O_2_ production in the acute exercise. This is further supported by a close correlation between p^66Shc^ and FOXO3a gene expressions and H_2_O_2_ content. Previous studies have shown that cells and tissues derived from p^66Shc^-null mice accumulate significantly less oxidative stress, and the deletion of p^66Shc^ gene in mice resulted in the decreased formation of mitochondrial H_2_O_2_ [16]. Our finding is also consistent with a latest study demonstrating that a prolonged swimming exercise promoted cellular oxidative stress and p^66Shc^ phosphorylation in rat heart [42] and supporting the hypothesis that acute exercise increases H_2_O_2_ levels involving upregulating p^66Shc^ signaling. p^66Shc^ is a peculiar protein, acting specifically in the mitochondrion as a redox enzyme that generates H_2_O_2_ by sequestering electrons from the respiratory chain [16]. It regulates H_2_O_2_ content through changes in H_2_O_2_ purification ability, membrane oxidase activity, and mitochondrial respiratory chain proton leak, so that levels of intracellular H_2_O_2_ maintain homeostasis in mammalian cells [17]. It has been known that a small fraction of p^66Shc^ translocates from the cytosol into the mitochondria, where it directly transfers electrons from cytochrome c to molecular oxygen, thus producing H_2_O_2_ [16]. Studies have demonstrated that PKC b, a protein kinase activated by oxidative stress, phosphorylated p^66Shc^ at serine 36, allowing its interaction with prolyl isomerase pin-1, which then physically translocated p^66Shc^ across the outer mitochondrial membrane [43]. Thus, oxidative stress-triggered p^66Shc^ phosphorylation and localization to mitochondria may play an important role in H_2_O_2_ generation during acute exercise. Our finding is similar to that of Ding et al. [12], who reported that acute exercise increased H_2_O_2_ content in triceps surae skeletal muscle in rats. In addition, there is a possibility that other metabolic and/or mitochondrial pathways may also be involved in regulation of H_2_O_2_ in the skeletal muscle during the acute exercise, as it has been shown that H_2_O_2_ generated by p^66Shc^ accounts for about 30% of the total pool of intracellular H_2_O_2_ [16]. Interestingly, previous studies have reported similar changes for other ROS species. For example, McArdle et al. found that aerobic contractile activity induced a release of superoxide anions from mouse gastrocnemius muscle* in vivo* [44]. One important observation in the present study is an association between H_2_O_2_ production and the downstream regulator FOXO3a, which supports the possibility that redox-dependent FOXO3a activation is regulated by intracellular H_2_O_2_ in a p^66Shc^-dependent manner during acute exercise. The possible link between H_2_O_2_ induced cell responses and FOXO3a was suggested previously [45]. Our finding is consistent with that by van der Horst et al. who showed that ROS induced a FOXO3a dependent antioxidant response [46, 47], indicating the p^66Shc^-H_2_O_2_-FOXO3a signalling interplay may play an important role in modulating cellular functions during exercise [48].

FOXO3a is a key transcription factor that translocates to nucleus and activates transcription by specifically binding to the consensus sequence TTGTTTAC in the promoters of target genes [49], causing an activation of transcription of the two essential antioxidant enzymes mitochondrial superoxide dismutase (MnSOD) and catalase, which scavenge superoxide and hydrogen peroxide, respectively [50]. It has been demonstrated that MnSOD and catalase are transcriptional targets of FOXO3a [51], and these enzymes are involved in the regulation of the cell cycle and the defence against oxidative stress [52]. Previous studies showed that increase in FOXO3a expression protected mitochondria dysfunction from hyperglycemia-induced oxidative stress in human lens epithelial cells [26]. FOXO3a reduced H_2_O_2_ -induced cellular oxidation and increased mitochondrial MnSOD protein expression (but no change in cytoplasmic copper/zinc SOD (CuZnSOD) expression) in DL23 cells [53] and controlled the expression of proteins involved in the DNA repair mechanisms [54]. In the present study, we found no significant changes of total SOD activity in all exercise groups; this finding is similar to a previous study which found no significant change of soleus muscle SOD activity by endurance exercise in rats [55]. On the contrary, Itoh et al. [56] reported a decrease in diaphragm SOD activity in rat with an acute exercise. Feoli et al. [57] also reported a decrease in serum SOD activity in volunteers with acute exercise. In addition, an increase of SOD activities in skeletal muscles and cardiac mitochondria after exercise has also been reported [58, 59]. The reason for these discrepancies is not clear, but it may be related to differences in exercise patterns, muscle types, and SOD assays used in these studies. For example, a 10-fold difference in the relative sensitivity among different SOD assays has been reported [60]. On the other hand, it is possible that FOXO3a may cause a change of a particular type of SOD activity (e.g, MnSOD), rather than total SOD activity [53]; thus further study is necessary to investigate the pattern of SOD changes and possible mechanisms in acute exercise. Similarly, the change of activity of catalase, another essential antioxidant enzyme targeted by FOXO3a target genes [17, 49] during the exercise, is also controversial. It was reported earlier that acute exercise significantly increased mitochondrial catalase activity, compared to the sedentary control [59]. Karanth and Jeevaratnam [55] also reported that swimming training increased muscle catalase content in rats. In contrast, Choi and Cho reported that catalase activity was significantly lower after 6 weeks of treadmill exercise [61]. It is not clear if this discrepancy related to the variations in catalase activities under different experimental conditions [62]. In the present study, we observed a slight decrease in catalase activity in the 90 min acute exercise group, then a significant increase in catalase activity in the 120 min and 150 min exercise groups, compared with that of the 90 min exercise group. It is possible that the initial decrease in the catalase activity is caused by the increased ROS production in mice with relative short period of exercise, and such decrease is corrected with the increase in FOXO3a-associated catalase enzymes [17] after longer period exercise. This seems to be in line with the observation that the slight decrease in catalase activity occurred earlier than the change of FOXO3a protein expression, while the increase in catalase activity occurred in the period with increased FOXO3a expressions. Further study with a longer period of exercise (e.g., >150 min) may help to elucidate FOXO3a-mediated increase in mitochondria catalase activity and related mechanisms. Thus, our findings indicate that mitochondrial catalase and FOXO3a related regulation of antioxidant enzymes may serve as important protective mechanisms in reducing acute exercise induced cellular oxidative stress.

In conclusion, our study demonstrates that acute exercise causes an increase in mitochondrial H_2_O_2_ production, which is associated with the upregulation of p^66Shc^ and FOXO3a in the skeletal muscles of ICR/CD-1 mice. Activation of the p^66Shc^-H_2_O_2_-FOXO3a signaling pathway by acute exercise may underline the molecular mechanism of regulating cellular oxidative stress resistance during exercise. Given that oxidative stress has been implicated in various diseases and aging, the mechanism behind this link may have therapeutic implications. A further understanding of the mechanism of acute exercise induced modulation of H_2_O_2_ production and associations to p^66Shc^ signaling and ROS-FOXO3a-antioxidant enzymes may help to develop interventions to improve exercise outcomes and control oxidative stress-related diseases or conditions such as diabetes and aging.
